# Melatonin increases growth and salt tolerance of *Limonium bicolor* by improving photosynthetic and antioxidant capacity

**DOI:** 10.1186/s12870-021-03402-x

**Published:** 2022-01-04

**Authors:** Junpeng Li, Yun Liu, Mingjing Zhang, Hualing Xu, Kai Ning, Baoshan Wang, Min Chen

**Affiliations:** 1 Shandong Provincial Key Laboratory of Plant Stress Research, College of Life Sciences, Shandong Normal University, Jinan, Shandong 250014 People’s Republic of China; 2 DongYing Academy of Agricultural Sciences, Dongying, Shandong 257000 People’s Republic of China

**Keywords:** Antioxidant, Gene expression, *Limonium bicolor*, Melatonin, Salt tolerance

## Abstract

**Background:**

Soil salinization is becoming an increasingly serious problem worldwide, resulting in cultivated land loss and desertification, as well as having a serious impact on agriculture and the economy. The indoleamine melatonin (N-acetyl-5-methoxytryptamine) has a wide array of biological roles in plants, including acting as an auxin analog and an antioxidant. Previous studies have shown that exogenous melatonin application alleviates the salt-induced growth inhibition in non-halophyte plants; however, to our knowledge, melatonin effects have not been examined on halophytes, and it is unclear whether melatonin provides similar protection to salt-exposed halophytic plants.

**Results:**

We exposed the halophyte *Limonium bicolor* to salt stress (300 mM) and concomitantly treated the plants with 5 μM melatonin to examine the effect of melatonin on salt tolerance. Exogenous melatonin treatment promoted the growth of *L. bicolor* under salt stress, as reflected by increasing its fresh weight and leaf area. This increased growth was caused by an increase in net photosynthetic rate and water use efficiency. Treatment of salt-stressed *L. bicolor* seedlings with 5 μM melatonin also enhanced the activities of antioxidants (superoxide dismutase [SOD], peroxidase [POD], catalase [CAT], and ascorbate peroxidase [APX]), while significantly decreasing the contents of hydrogen peroxide (H_2_O_2_), superoxide anion (O_2_^•−^), and malondialdehyde (MDA). To screen for *L. bicolor* genes involved in the above physiological processes, high-throughput RNA sequencing was conducted. A gene ontology enrichment analysis indicated that genes related to photosynthesis, reactive oxygen species scavenging, the auxin-dependent signaling pathway and mitogen-activated protein kinase (*MAPK*) were highly expressed under melatonin treatment. These data indicated that melatonin improved photosynthesis, decreased reactive oxygen species (ROS) and activated MAPK-mediated antioxidant responses, triggering a downstream MAPK cascade that upregulated the expression of antioxidant-related genes. Thus, melatonin improves the salt tolerance of *L. bicolor* by increasing photosynthesis and improving cellular redox homeostasis under salt stress.

**Conclusions:**

Our results showed that melatonin can upregulate the expression of genes related to photosynthesis, reactive oxygen species scavenging and mitogen-activated protein kinase (*MAPK*) of *L. bicolor* under salt stress, which can improve photosynthesis and antioxidant enzyme activities. Thus melatonin can promote the growth of the species and maintain the homeostasis of reactive oxygen species to alleviate salt stress.

**Supplementary Information:**

The online version contains supplementary material available at 10.1186/s12870-021-03402-x.

## Background

Biotic and abiotic stresses severely inhibit plant growth and development [38, 52]. Among these stressors, soil salinization is becoming an increasingly serious problem worldwide, causing cultivated land loss and desertification and seriously impacting agriculture and the economy [10, 17, 18, 36]. Some plants such as halophytes with high salt tolerance can survive in saline soils being higher or equal to 200 mM NaCl, which can increase the use of saline land and even improve it [7, 11, 31, 32, 41]. Salt stress causes not only osmotic and ionic stresses, but also oxidative stress, which inhibits photosynthesis, nutrient use, protein synthesis, and enzyme activity [8, 17, 18, 37]. Moreover, oxidative stress caused by high salt stress leads to the accumulation of large amounts of reactive oxygen species (ROS), such as hydrogen peroxide (H_2_O_2_) and superoxide anion (O_2_^•–^) [25, 40]. The excessive ROS inhibit photosynthesis, respiration, and protein synthesis and disrupt membrane structure, etc. [16, 19, 35], thereby inhibiting growth and, in severe cases, causing death. To overcome oxidative stress, plants can upregulate antioxidative enzymes activities and increase nonenzymatic antioxidants levels [25]. The antioxidative enzymes in plants consist mainly of superoxide dismutase (SOD), peroxidase (POD), catalase (CAT), and ascorbate peroxidase (APX) (Apel et al. 2004 [28];). In addition, low level of ROS act as second messengers that activate stress-related regulators, such as mitogen-activated protein kinase (MAPK) and transcription factors, which activate the expression of stress-related genes and enhance plant tolerance [15]. The activation of a plant’s antioxidant system under adverse conditions helps the plant maintain its normal life activities and increases its tolerance to stress.

Photosynthesis, the plant’s source of energy and fixed carbon, consists of light and dark reactions [8]. Salt stress decreases the supply of CO_2_ and inhibits the activity of enzymes involved in the dark reactions, which diverts excess light energy from the light reactions into ROS production [15, 26]. Excessive ROS in turn disrupt thylakoid membrane structure and reduce the activity of light reaction enzymes, resulting in a decline in photosynthesis. Therefore, the timely removal of ROS produced under salt stress is essential for the maintenance and restoration of photosynthesis.

Melatonin (N-acetyl-5-methoxytryptamine) was first identified in animals, where it regulates circadian sleep rhythms [20] and the immune system [1, 50] and increases the activities of antioxidant enzymes [34]. Melatonin has multiple physiological functions in regulating plant growth and developmental processes, such as melatonin significantly enhanced the numbers of adventitious roots of *Solanum lycopersicum* [47], delayed leaves senescence of apple [46] etc. In addition, studies have shown that melatonin can increase plant resistance to various stresses. Liu et al. [27] showed that *SlCOMT1*, a melatonin biosynthesis-related gene from tomato (*Solanum lycopersicum* Mill. cv. *Ailsa Craig*), overexpression transgenic tomato plants had higher melatonin content than wild-type plants, which can improve *SlCOMT1* overexpression plants resistance to salt stress. Zhu et al. [57] showed that exogenous melatonin significantly improved *Arabidopsis thaliana* resistance to *Botrytis cinerea* infection by enhancing activities of antioxidative enzymes and maintaining intracellular H_2_O_2_ concentrations at steady-state levels. Salt stress inhibit the growth of Alfalfa (*Medicago sativa* L.) and melatonin significantly alleviated this growth inhibition by reducing ROS content [2]. Under salt stress, melatonin can induce lateral root formation of cucumber (*Cucumis sativus* L.) seedlings and RNA sequencing showed that peroxidase-related genes to be involved in the melatonin response [56]. Sun et al. [42] showed that overexpression of *COMT1* (Caffeic Acid O-Methyltransferase 1) in tomato (*Solanum lycopersicum*) significantly increased melatonin level and salt stress tolerance of transgenic lines by improving antioxidant capacity. Although some studies report that melatonin enhances the salt tolerance of glycophytes [2, 42, 57], to the best of our knowledge, studies on melatonin-induced salt tolerance of halophyte are elusive.

Halophyte can grow well in moderately saline soil while whose growth is inhibited under high salt stress. *Limonium bicolor* is a typical secretohalophyte with epidermal salt glands. This species grows best under moderately salty conditions (100 mM NaCl), but its growth is inhibited by high salt stress [9, 53]. *L. bicolor* is used in traditional Chinese medicine and has excellent medicinal value for treating blood disorders, heat-evil, hepatitis, diarrhea, bronchitis, emmeniopathy, uterine cancer, and other diseases (Li 1978). However, the growth inhibition of *L. bicolor* under high salt conditions has restricted the effective use and development of the species. Our previous results showed that exogenous melatonin can improve the growth of *L. bicolor* under salt stress by increasing salt secretion capacity of salt gland [24]. In addition to increasing its salt secreting ability, whether exogenous melatonin can be through other ways to relieve the high salt stress of the species? In this study, we examined the effect of exogenous melatonin on growth, photosynthesis, and antioxidant enzyme activity in *L. bicolor*. These results provide a basis for further studying the melatonin response mechanism of plants, especially of halophytes under salt stress.

## Results

### Melatonin promotes *L. bicolor* growth under salt stress

**Fig. 1:**
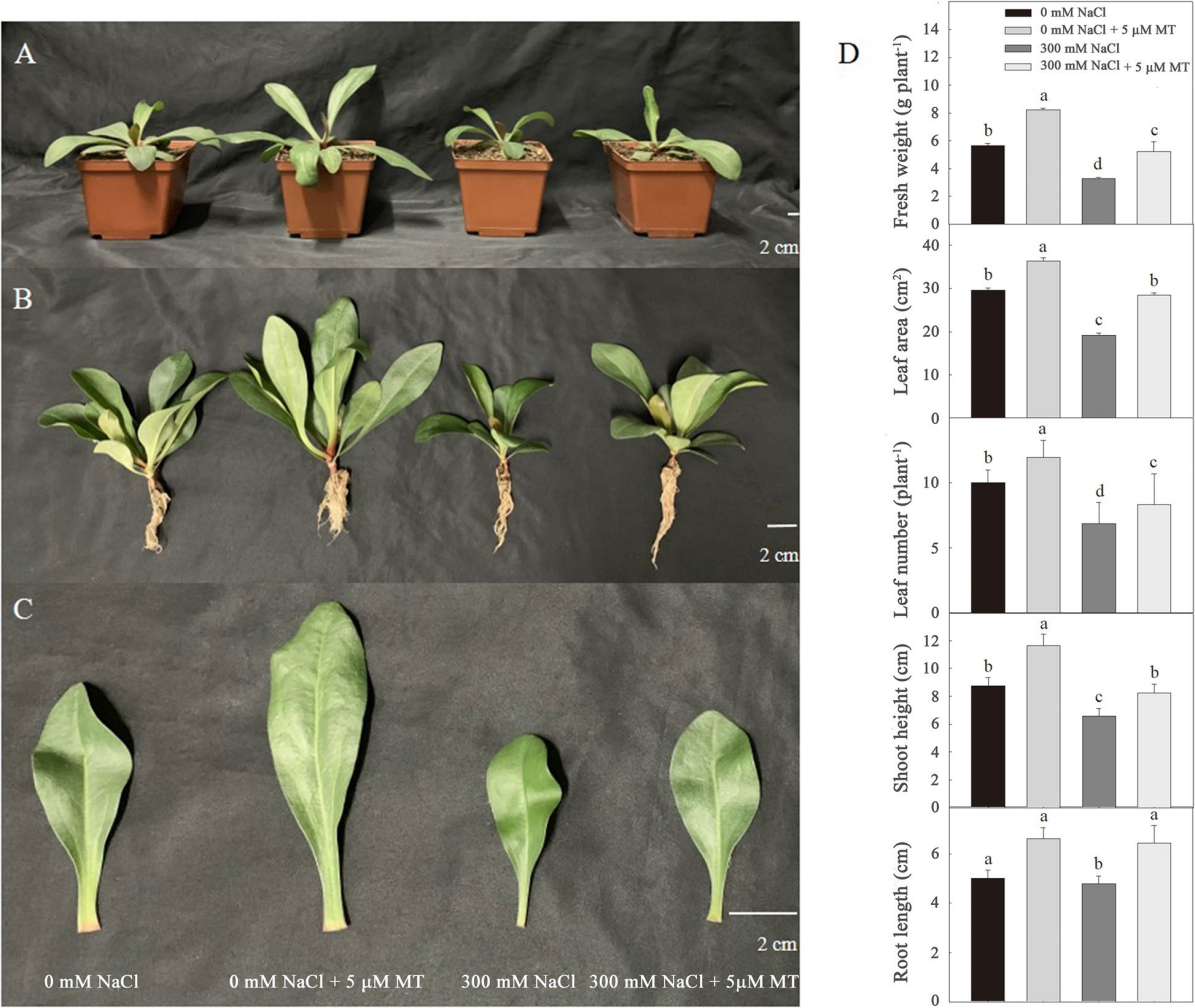
Effects of melatonin (5 μM) on (**A-B**) the overall growth, (**C**) leaf size and (**D**) fresh weight, leaf area, leaf number, shoot height and root length of *L. bicolor* seedlings subjected to 300 mM NaCl for 15 days. Values are mean ± standard deviation of five biological replicates. Bars labeled with different letters are significantly different at *P* < 0.05 according to Duncan’s multiple range tests. 0 mM NaCl, plants cultivated with only Hoagland nutrient solution; 0 mM NaCl + 5 μM MT, plants cultivated with Hoagland nutrient solution plus 5 μM melatonin; 300 mM NaCl, plants cultivated with Hoagland nutrient solution plus 300 mM NaCl; 300 mM NaCl + 5 μM MT, plants cultivated with Hoagland nutrient solution plus 300 mM NaCl and 5 μM melatonin

Salt stress (300 mM NaCl) retarded growth rate of *L. bicolor* seedlings, as evidenced by the reduction of fresh weight, shoot height, root length (Fig. 1A, B and D), leaf area (Fig. 1C, D) and the number of leaves (Fig. 1D). Exogenous melatonin treatment (5 μM) improved the growth of *L. bicolor* seedlings, resulting in a greater fresh weight and leaf area, higher shoot height, longer root length and more leaves than those of control seedlings, regardless of whether the seedlings were treated with 0 mM or 300 mM NaCl (Fig. 1). After 15 days of treatment, seedlings treated with exogenous melatonin and 0 mM NaCl had a fresh weight that was 49.1% greater than that of untreated control seedlings, whereas those treated with both melatonin and 300 mM NaCl had a fresh weigh that was 64.5% greater than that of seedlings treated with 300 mM NaCl only.

### Melatonin increases chlorophyll content and photosynthesis of *L. bicolor* under salt stress

**Fig. 2:**
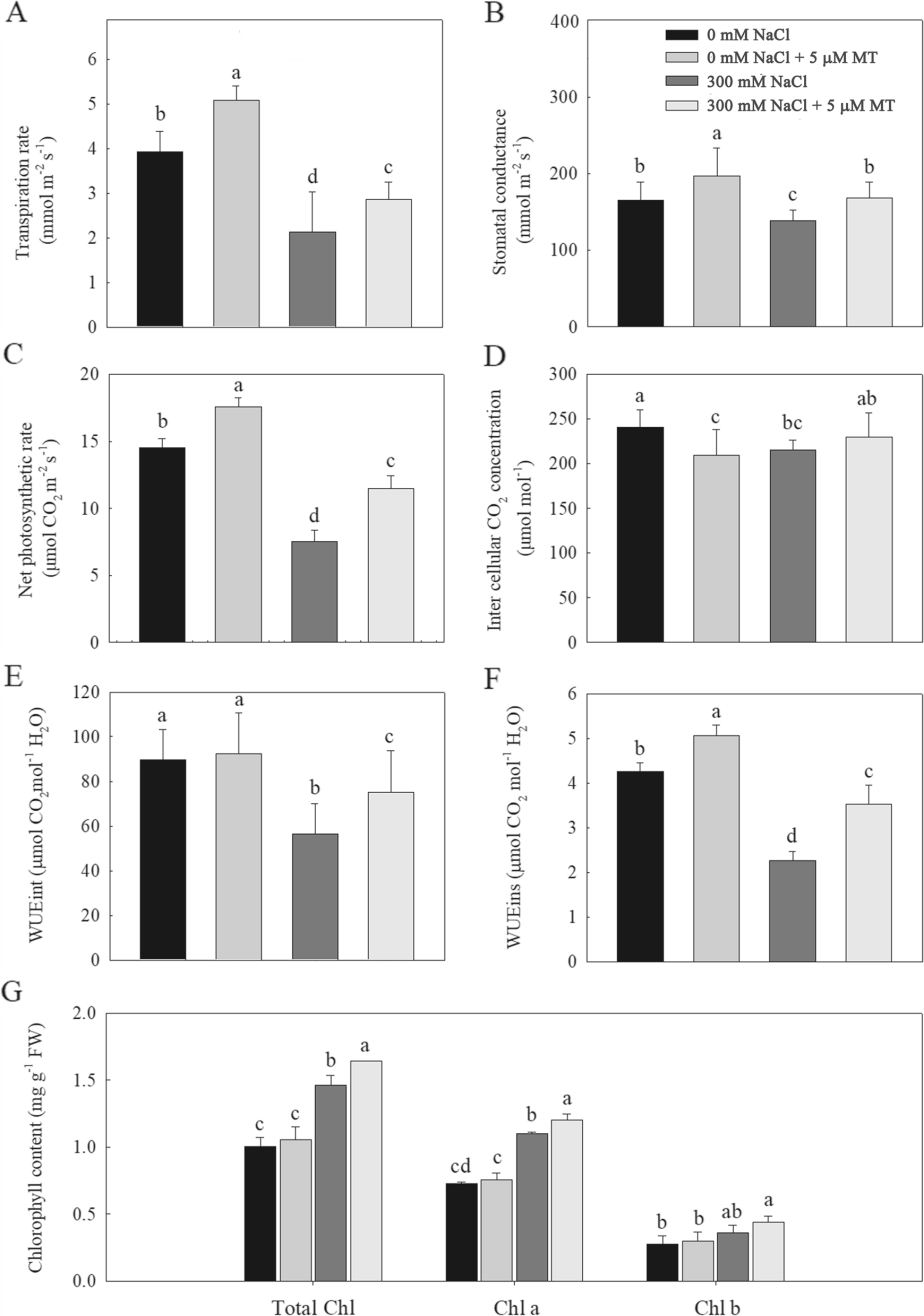
Effects of melatonin (5 μM) on (**A**) transpiration rate (Tr), (**B**) stomatal conductance (g_s_), (**C**) net photosynthetic rate (Pn), (**D**) inter cellular CO_2_ concentration (Ci), (**E**) intrinsic water use efficiency (WUEint), (**F**) instantaneous water use efficiency (WUEins), (**G**) total chlorophyll, chlorophyll a, chlorophyll b content of *L. bicolor* seedlings subjected to 300 mM NaCl for 15 days. Values are mean ± standard deviation of five biological replicates. Bars labeled with different letters are significantly different at *P* < 0.05 according to Duncan’s multiple range tests. Chl, Chlorophyll

*L. bicolor* seedlings treated with salt for 15 days had considerably lower transpiration rates (Tr, Fig. 2A), stomatal conductance (g_s_, Fig. 2B), net photosynthetic rate (Pn, Fig. 2C), intercellular carbon dioxide concentration (Ci, Fig. 2D), intrinsic water use efficiency (WUEint, Fig. 2E) and instantaneous water use efficiency (WUEins, Fig. 2F) than untreated controls. Application of exogenous melatonin for 15 days led to significantly increased Tr, g_s_, Pn, WUEint and WUEins in both NaCl-treated and untreated plants.

Total chlorophyll, chlorophyll a, and chlorophyll b (Fig. 2G) contents of *L. bicolor* seedlings significantly increased under treatment with 300 mM NaCl compared to those of controls. Total chlorophyll and chlorophyll a contents increased even further in salt-stressed plants treated with exogenous melatonin.

### Melatonin reduces oxidative stress in *L. bicolor* under salt stress

**Fig. 3:**
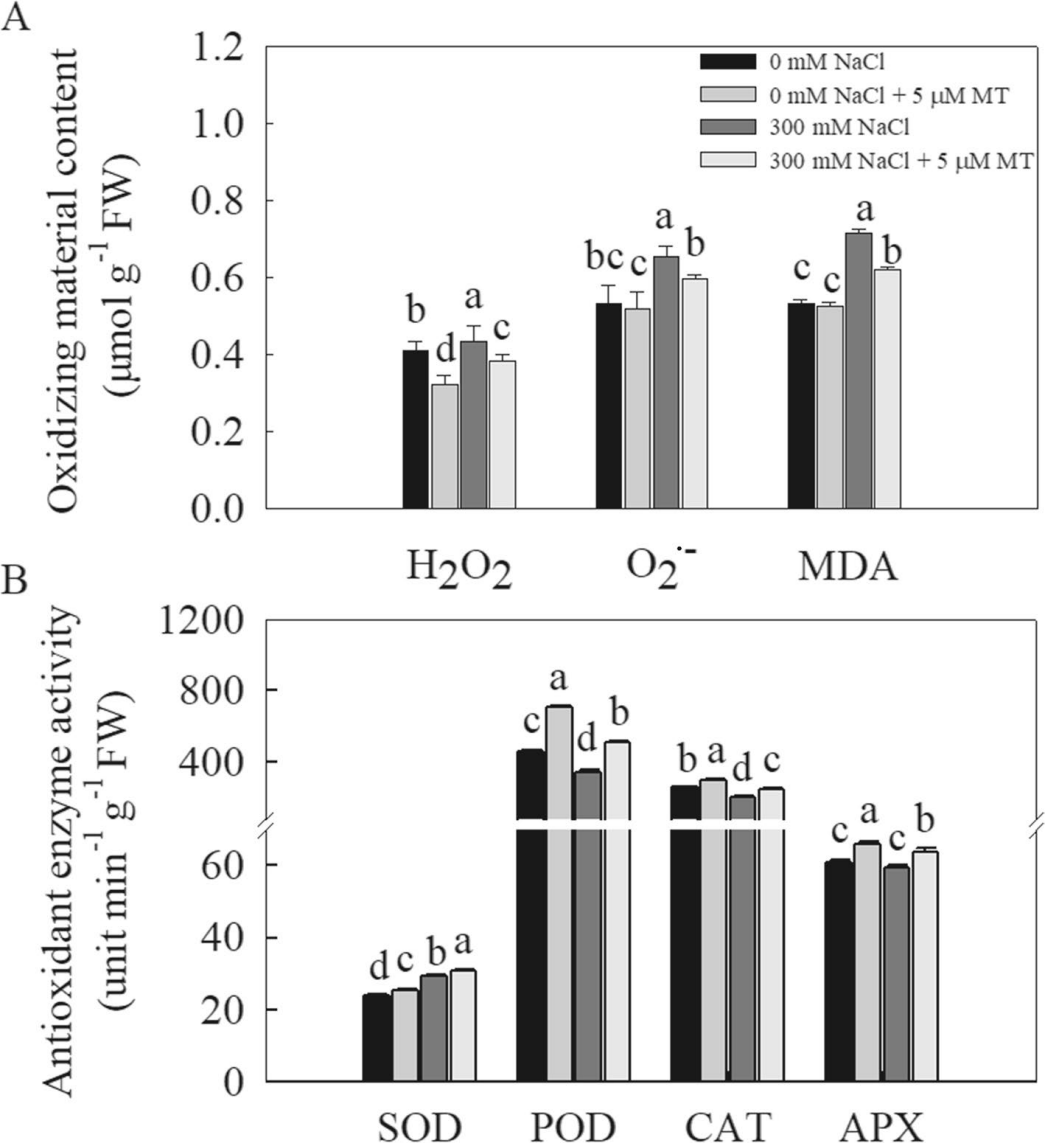
Effects of melatonin (5 μM) on (**A**) H_2_O_2_ content, O_2_^•–^ content and MDA content, (**B**) Antioxidant enzymes (SOD, POD, CAT and APX) activities of *L. bicolor* seedlings subjected to 300 mM NaCl for 15 days. Values are mean ± standard deviation of five biological replicates. Bars labeled with different letters are significantly different at *P* < 0.05 according to Duncan’s multiple range tests. 0 mM NaCl, plants cultivated with only Hoagland nutrient solution; 0 mM NaCl + 5 μM MT, plants cultivated with Hoagland nutrient solution plus 5 μM melatonin; 300 mM NaCl, plants cultivated with Hoagland nutrient solution plus 300 mM NaCl; 300 mM NaCl + 5 μM MT, plants cultivated with Hoagland nutrient solution plus 300 mM NaCl and 5 μM melatonin

The ROS produced under salt stress consist mainly of H_2_O_2_ and O_2_^•–^. The H_2_O_2_ and O_2_^•–^ levels in the *L. bicolor* seedlings significantly increased under salt stress, whereas melatonin treatment significantly attenuated these increases (Fig. 3A). Similarly, MDA content, which reflects the degree of cell membrane damage, significantly increased under NaCl stress, whereas melatonin treatment significantly decreased MDA content in the salt-stressed seedlings (Fig. 3A).

### Melatonin increases the activities of antioxidant enzymes in *L. bicolor* under salt stress

Plants have developed antioxidant systems that remove ROS and help prevent the cellular damage induced by excess ROS under unfavorable conditions. Antioxidant enzymes play an important role in removing excess ROS. A 300 mM NaCl treatment significantly increased SOD activity, decreased the activities of POD and CAT, and had no significant effect on APX activity (Fig. 3B) in *L. bicolor* seedlings. However, exogenous melatonin treatment significantly increased SOD (increased by 14.3%), POD (42.9%), CAT (25.0%), and APX (8.6%) activities compared to salt-stressed seedlings without melatonin.

### Melatonin upregulates the expression of genes related to photosynthesis and reactive oxygen species scavenging in *L. bicolor* under salt stress

**Fig. 4:**
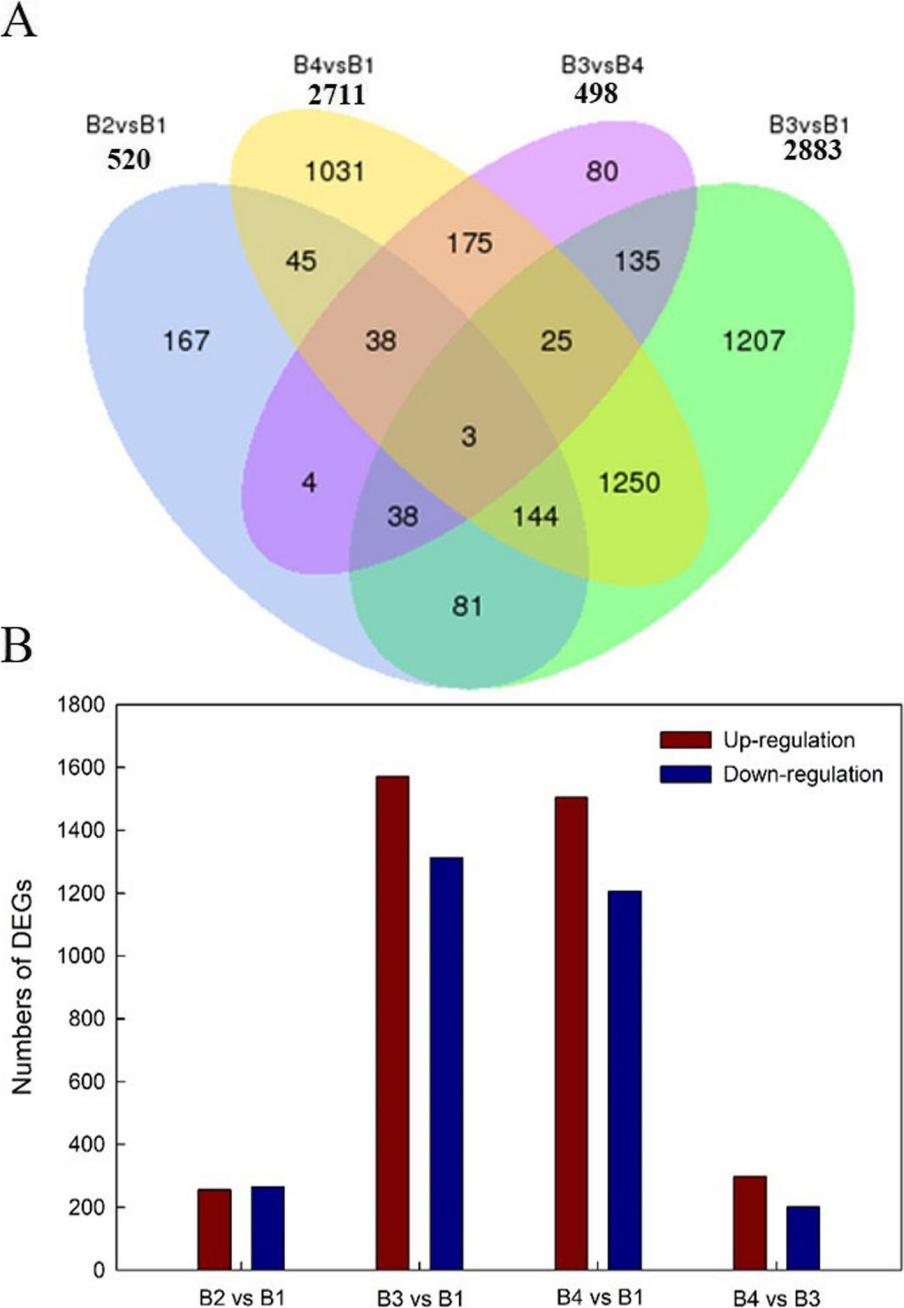
Effects of melatonin (5 μM) on genes expression of *L. bicolor* seedlings subjected to 300 mM NaCl for 24 h. (A) Venn diagram showing numbers of overlapping DEGs in the transcriptome data under different treatment, (B) Numbers of DEGs in the transcriptome data of *L. bicolor* seedlings  treated with 300 mM NaCl for 24 h

To investigate the mechanism underlying melatonin-induced NaCl stress tolerance in *L. bicolor* seedlings, dynamic profiling of the mRNA expression was obtained via transcriptome sequencing. Totally, 520 (256 upregulated genes and 264 downregulated genes), 2883 (1571 upregulated genes and 1312 downregulated genes), 2711 (1501 upregulated genes and 1206 downregulated genes) and 498 (297 upregulated genes and 201 downregulated genes) differently expressed genes (DEGs) were separately identified in contrastive groups of ‘B2 vs. B1’, ‘B3 vs. B1’, ‘B4 vs. B1’ and ‘B4 vs. B3’ (Fig. 4). Most numbers of DEGs were shown in NaCl vs. control, which involved in ions transporters and salt secretion from the salt gland and had been confirmed before (Li et l., 2020). About 500 DEGs were shown in ‘melatonin vs. control’ or ‘melatonin+NaCl vs. NaCl’, which suggested that melatonin may involve in regulating genes expression in *L. bicolor*. Venn diagram analysis showed that 83 DEGs were influenced by melatonin under both control and NaCl stress conditions (the intersection of “B2 vs. B1’ and ‘B4 vs. B3’), which implied that these genes might be involved in melatonin-induced NaCl stress tolerance.

**Fig. 5:**
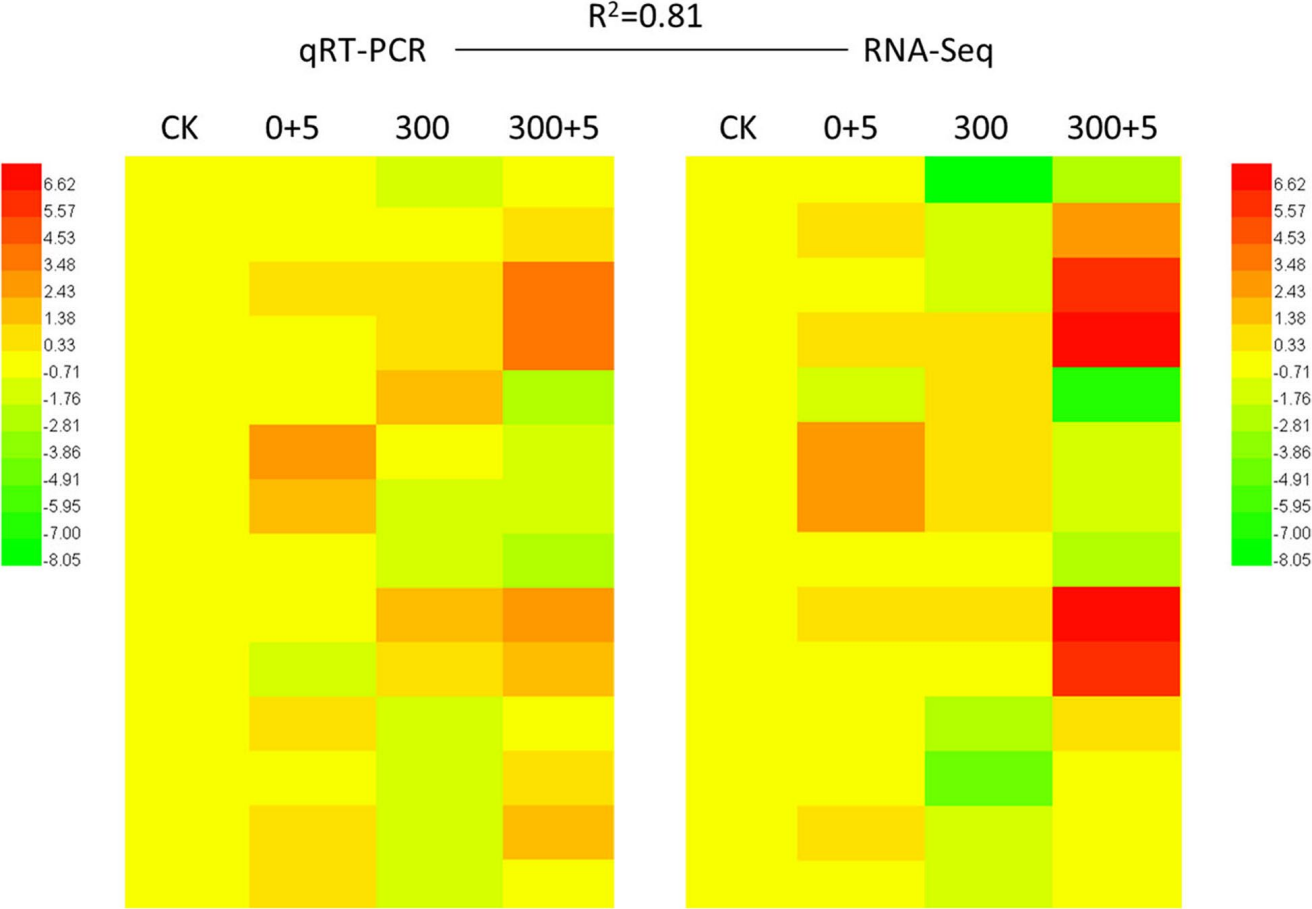
Validation of RNA-Seq results by qRT-PCR using 14 *L. bicolor* candidate genes. The expression levels of 14 randomly selected genes under different treatment are shown. A red color indicates that the gene is highly expressed under the corresponding treatment. CK (0 mM NaCl), plants cultivated with only Hoagland nutrient solution; 0+5 (0 mM NaCl + 5 µM MT), plants cultivated with Hoagland nutrient solution plus 5 μM melatonin; 300 (300 mM NaCl), plants cultivated with Hoagland nutrient solution plus 300 mM NaCl; 300+5 (300 mM NaCl + 5 µM MT), plants cultivated with Hoagland nutrient solution plus 300 mM NaCl and 5 μM melatonin

Most of the 83 DEGs influenced by melatonin under both control and NaCl involved in photosynthesis, reactive oxygen species scavenging, IAA (indoleacetic acid) function and ion transport in *L. bicolor* (Table 1). To validate the correlation between the RNA-seq and qRT-PCR results, we designed 14 pairs of primers (Table S1) to target the DE gene transcripts. The tested genes encode proteins that are involved in photosynthesis, ROS scavenging system and ion transport. The results from the RNA-seq and qRT-PCR showed that the correlation between the transcriptome sequencing and qRT-PCR values was high (R^2^ = 0.81, Fig. 5).

**Table 1:** Differentially expressed and highly transcribed genes related to genes related to photosynthesis and reactive oxygen species scavenging in the different treatment *Limonium bicolor* leaves

Gene ID	B1	B2	FC	B3	B4	FC
ADG1: Encodes the small subunit of ADP-glucose pyrophosphorylase.						
Lb0G37377	612.33	732.32	0.26 up	29.54	644.96	4.45 up
DGD2: Encodes a UDP-galactose-dependent digalactosyldiacylglycerol (DGDG) synthase.						
Lb1G03486	96.50	1.96	−5.62 down	2.91	82.79	4.83 up
ATSUC4: Low affinity (10 mM) sucrose transporter in sieve elements (phloem).						
Lb1G06992	4.05	65.24	4.01 up	4.28	121.09	4.82 up
Lb3G15617	24.60	NS	NS	2.46	52.38	4.41 up
Phosphofructokinase family protein.						
Lb6G30828	22.58	201.71	3.16 up	20.99	361.12	4.10 up
PNAD-MDH: Encodes a protein with NAD-dependent malate dehydrogenase activity.						
Lb1G04420	826.21	843.68	0.03 up	140.65	1082.78	2.94 up
PSBA: Encodes chlorophyll binding protein D1.						
Lb0G36726	52.91	48.85	−0.12 down	31.05	4660.16	7.23 up
Lb4G25364	5.29	7.53	0.51 up	10.30	1219.19	6.89 up
Lb0G37372	77.23	82.87	0.10 up	85.21	4367.29	5.68 up
RRN16S.2: Chloroplast-encoded 16S ribosomal RNA.						
Lb0G37563	15.63	13.01	−0.26 down	10.34	1074.28	6.69 up
Lb4G25504	12.09	16.79	0.47 up	16.42	1754.74	6.74 up
CRR42: Chloroplast NADH dehydrogenase assembly protein.						
Lb3G18424	410.01	460.86	0.17 up	11.66	172.86	3.89 up
ATPA: Encodes the ATPase alpha subunit.						
Lb4G23216	3.39	4.98	0.55 up	2.21	231.44	6.71 up
DALL5: Encodes a triacylglycerol lipase located in plastoglobuli.						
Lb2G08239	3.00	39.17	3.70 up	2.47	31.23	3.66 up
RLK7: RLK7 belongs to a leucine-rich repeat class of receptor-likekinase (LRR-RLKs).						
Lb4G23386	3.39	4.98	0.55 up	2.21	231.44	6.71 up
ABO8: Encodes ABO8, a pentatricopeptide repeat (PPR) protein.						
Lb5G26864	26.36	57.19	1.12 up	10.52	99.57	3.24 up
NAD (P)-linked oxidoreductase superfamily protein.						
Lb6G30054	26.36	57.19	1.12 up	10.52	99.57	3.24 up
ATGPX6: Encodes glutathione peroxidase.						
Lb0G37493	298.78	536.27	0.84 up	200.86	711.49	1.82 up
HPCA1: Leucine rich receptor kinase.						
Lb3G15760	399.21	115.54	−1.79 down	93.39	381.65	2.03 up
DCC1: Encodes a putative thioredoxin DCC1.						
Lb2G08796	NS	NS	NS	2.78E-17	25.01	59.64 up
AO: Role in the degradation of ascorbate to (mono) dehydroascorbate.						
Lb1G05941	NS	88.01	NS	7.87	77.58	3.30 up
MSRB1: 1-Cys methionine sulfoxide reductase.						
Lb3G20002	26.25	24.76	−0.08 down	26.27	316.42	3.59 up
MAD3: Encodes a 3-hydroxy-3-methylglutaryl coenzyme A reductase.						
Lb3G18131	9.18	139.08	3.92 up	9.18	67.49	2.88 up
PRX17: Encodes a cell wall-localized class III peroxidase.						
Lb0G37582	10.60	72.48	2.77 up	21.13	126.40	2.58 up
DSPTP1E: Encodes a protein phosphatase that interacts with MPK12.						
Lb0G38281	9.09	124.93	3.78 up	9.18	67.49	2.88 up
FQR1: Encodes a flavin mononucleotide-binding flavodoxin-like quinone reductase.						
Lb1G00304	1483.82	1629.17	0.13 up	574.94	1387.82	1.27 up
GH3.1: Encodes a protein similar to IAA-amido synthases.						
Lb3G17391	38.75	35.25	−0.14 down	35.82	186.15	2.38 up
PGP21: Encodes a facultative transporter controlling auxin concentrations in plant cells.						
Lb3G19218	33.44	NS	NS	89.32	319.35	1.84 up

Differentially expressed genes were filtered using the threshold of Informative/Non-Informative (I/NI) C 0.4 using the Dexus method. “FC (Fold change)” equals log_2_ (B2-FPKM/B1-FPKM or B4-FPKM/B3-FPKM), “up” represents up-regulated genes and “down” represents down-regulated genes. B1, 0 mM NaCl+ 0 μM melatonin; B2, 0 mM NaCl+ 5 μM melatonin; B3, 300 mM NaCl+ 0 μM melatonin; B1, 300 mM NaCl+ 5 μM melatonin

**Fig. 6:**
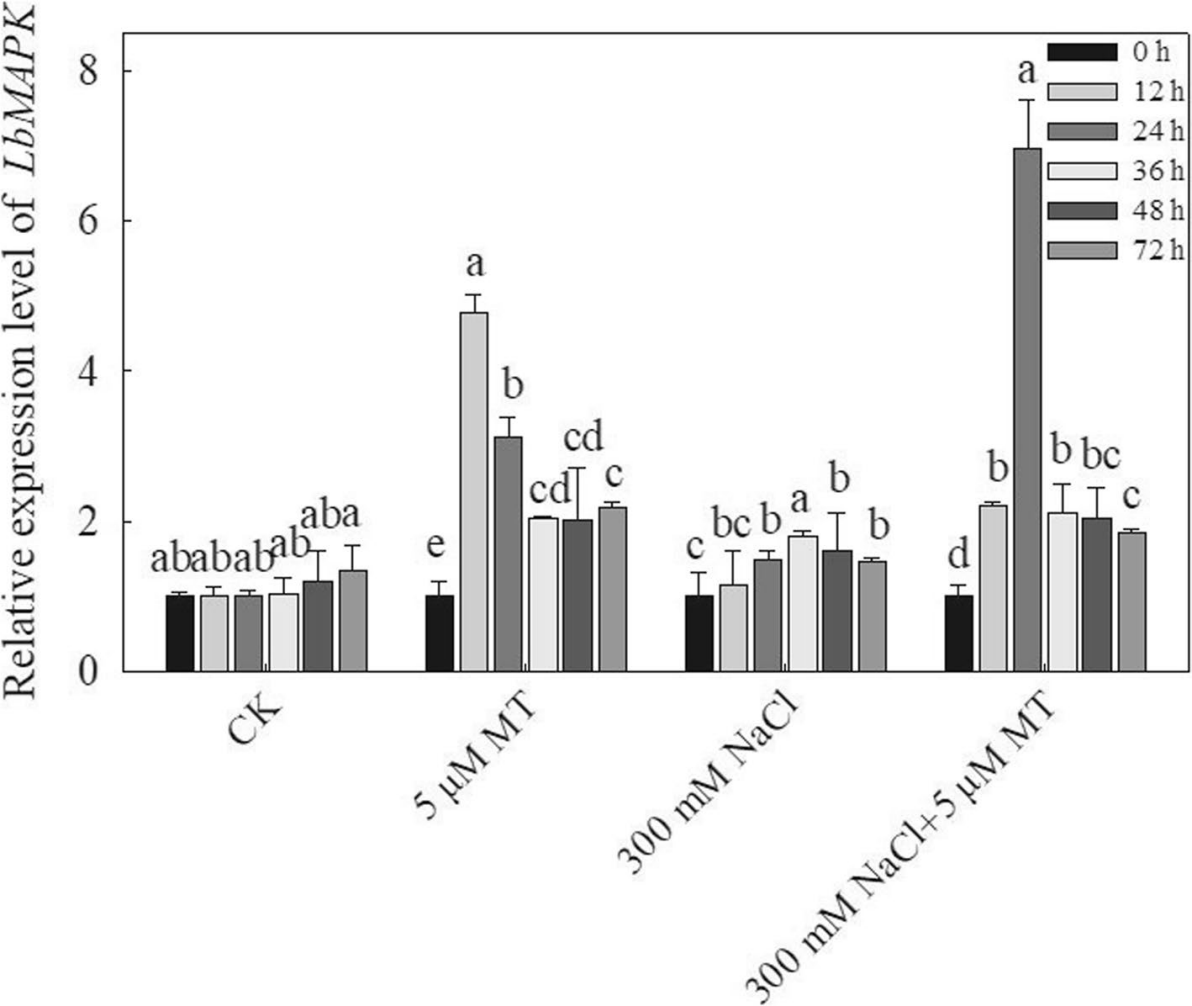
Effects of melatonin (5 μM) on *MAPK* gene expression of *L. bicolor* leaves in six-week-old seedlings subjected to 300 mM NaCl for 0, 12, 24, 48 and 72 h. Values are mean ± standard deviation of three biological replicates. Bars labeled with different letters are significantly different at *P* < 0.05 according to Duncan’s multiple range tests. CK, 0 mM NaCl

Go (gene ontology) analysis showed that melatonin upregulated the genes expression related to MAPK signaling pathways, photosynthetic structure and membrane, ion channels or transporters and some metabolic process etc. under salt stress (Fig. S1). In the upregulated genes, *MAPK3* expression was examined under melatonin and salt treatment in the species. The results showed that 300 mM NaCl treatment for 24–72 h significantly upregulated the gene expression, while melatonin treatment for 12–72 h significantly upregulated the gene expression regardless of whether the seedlings were treated not or with 300 mM NaCl. Compared to the control, the gene expression upregulated 4.6 times under melatonin treatment for 12 h, while 6.7 times under melatonin and 300 mM NaCl treatment for 24 h (Fig. 6).

## Discussion

Melatonin has received increasing attention in studies of plant stress tolerance [3, 39]. Previous studies showed that exogenous melatonin application can alleviate salt-induced growth inhibition in some non-halophytes, such as watermelon (*Citrullus lanatus*) and cucumber [22, 45, 49]. However, whether exogenous melatonin treatment can alleviate salt-induced growth inhibition in halophytes was rarely reported. Our previous results showed that exogenous melatonin can increase salt secretion from salt glands of secretohalophyte *L. bicolor*, decrease the content of Na^+^ and Cl^−^ in leaves to alleviate NaCl stress [24]. Whether melatonin alleviate NaCl-induced growth inhibition to the species through other ways? In this study, we treated *L. bicolor* seedlings with salt and melatonin and showed that 5 μM melatonin promotes plant growth under salt stress, increasing the fresh weight and leaf area of the treated plants (Fig. 1). The melatonin-induced increase in biomass was associated with enhanced photosynthesis. NaCl stress inhibited photosynthesis in *L. bicolor* seedlings [52], whereas pretreatment with melatonin significantly increased photosynthesis (Fig. 2), which is consistent with previous results in nonhalophytes [22, 45]. Photosynthesis is a key physiological process that is sensitive to salt stress [43]. Treatment of *L. bicolor* with 300 mM NaCl resulted in stomatal closure and thus a decrease in stomatal conductance, which also led to decreases in transpiration from the leaves, intercellular CO_2_ concentration, net photosynthetic rate and water use efficiency (Fig. 2). Exogenous melatonin application can promote stomatal opening and improve stomatal function (Fig. 2c), thereby increasing intercellular CO_2_ concentrations, water-use efficiency and improving net photosynthetic efficiency. This result is consistent with previous observations in watermelon ([22, 33]; Rahman et al. 2020). Although salt stress inhibited photosynthesis in *L. bicolor* seedlings, it led to significantly increased levels of chlorophyll in the leaves (Fig. 2a). This result showed that, at least for this species, salt stress inhibited photosynthesis not through a reduction of chlorophyll content but through stomatal factors [22]. Exogenous melatonin treatment increased photosynthesis in *L. bicolor* by improving stomatal function and enabling the plants to reopen their stomata under salt stress [46].

The osmotic stress associated with salt stress often causes dehydration and leads to ROS production [23, 51]. In addition, salt stress inhibits photosynthetic CO_2_ assimilation, which leads to the accumulation of excess light energy and the production of even more ROS [13]. ROS can cause serious oxidative damage to lipids, proteins, and nucleic acids, thereby damaging cells and disrupting plant metabolism [12]. Under salt stress, plants accumulate large amounts of ROS, such as peroxides, hydroxyl groups, and peroxy radicals, leading to oxidative stress, which in turn leads to increased levels of malondialdehyde (MDA), membrane dysfunction, and even cell death [21]. Consistent with these observations from other plants [2, 15, 21], the amounts of H_2_O_2_, O_2_^•–^, and MDA increased significantly in *L. bicolor* under salt stress (Fig. 3a, b). Exogenous melatonin treatment can reduce the contents of ROS (such as H_2_O_2_ and O_2_^•–^) and MDA in salt-stressed *L. bicolor*. Melatonin is a well-known antioxidant that directly removes ROS and stabilizes the ROS concentration [4]. In addition, melatonin can increase antioxidant enzyme activity during oxidative stress [15]. Under salt stress, the activities of antioxidant enzymes (such as SOD, POD, CAT, and APX) in *L. bicolor* significantly increased after melatonin was exogenously applied (Fig. 3). The increased activities of these enzymes could remove excessive ROS and improve cellular redox homeostasis under salt stress [44, 45].

In eukaryotes, the MAPK signaling pathways play crucial roles in regulating plant responses to salt stress [30]. Studies showed that MAPK components can enhance salt stress tolerance by increased antioxidant enzyme activities [29, 55]. In this study, melatonin can activate the MAPK signal cascade, regulate the expression of antioxidant enzyme genes, promote the activity of antioxidant enzymes, and ultimately enhance the tolerance of plants to abiotic stresses, which is consist to Xia et al. [48]. Salt stress increased the expression level of the *MAPK* gene in *L. bicolor*, and *MAPK* expression increased significantly after the application of exogenous melatonin (Fig. 6). Melatonin probably induced the MAPK cascade through the H_2_O_2_ pathway [47], which would further enhance the salt tolerance of *L. bicolor* seedlings. These results are consistent with previous results in naked oat (*Avena nuda* [14];).

In summary, transcriptome sequencing has been used to investigate the mechanism underlying melatonin alleviate NaCl-induced growth inhibition by improving photosynthesis and increasing antioxidant enzyme activities. Exogenous melatonin can upregulate genes expression related to photosynthesis and reactive oxygen species scavenging, which can promote growth and alleviate oxidative stress caused by salt stress. Melatonin acts as a strong antioxidant that directly scavenges ROS and also removes ROS by enhancing other antioxidant enzyme activities [44]. Furthermore, melatonin treatment induces the expression of *MAPKs*, thereby regulating the expression of downstream stress-responsive genes and improving the salt tolerance of *L. bicolor*.

## Materials and methods

### Plant materials and growth conditions

*L. bicolor* seeds were kindly provided by Professor Xu Hualing, Dongying Academy of Agricultural Sciences, Shandong Province. The seeds were sterilized according to Li et al. [23] and then sown on well-washed river sand in plastic pots (16 cm in diameter), which were placed in a growth chamber with 600 μmol m^− 2^ s^− 1^ light (15-h day/9-h night photoperiod), a temperature of 25 ± 3 °C/22 ± 3 °C (day/night), and a relative humidity of 60/80% (day/night). After the leaves emerged, the plants were watered with Hoagland’s nutrient solution.

### Combined NaCl and melatonin treatment

When the seedlings reached the six-leaf stage (one-month-old seedlings), they were subjected to NaCl and melatonin treatments. For the NaCl treatment, the seedlings were treated with Hoagland’s nutrient solution containing NaCl, which was increased by 50 mM every 12 h to a final concentration of 300 mM to avoid salt shock. The control seedlings were treated with Hoagland’s nutrient solution only. To examine the effects of melatonin on *L. bicolor* seedlings, the NaCl-treated and control seedlings were irrigated with 0 or 5 μM melatonin dissolved in Hoagland’s nutrient solution. The experiment for transcriptome included four treatments: (i) control (marked as: B1), *L. bicolor* plants cultivated with only Hoagland nutrient solution; (ii) melatonin (marked as: B2), Hoagland nutrient solution plus 0.5 μM melatonin; (iii) NaCl stress (marked as: B3), Hoagland nutrient solution plus 300 mM NaCl; (iv) NaCl stress with melatonin (marked as: B4) Hoagland nutrient solution plus 300 mM NaCl and 0.5 μM melatonin. The *L. bicolor* seedlings were treated with various combinations of salt and melatonin every 12 h for 15 consecutive days. Five replicates (3 plants per replicate) were used for each treatment. After 15 days, the leaves were collected to determine the biological indicators.

### Photosynthetic index, water use efficiency and chlorophyll content measurements

After exposure to NaCl and melatonin treatment for 15 days, photosynthetic parameters were measured in fully expanded leaves using a Li-6000 portable photosynthesis measurement system (LI-COR, Inc., Lincoln, NE, USA). The measurements were performed from 10:00 AM to 12:00 AM and five replicates were performed per treatment.

The parameters intrinsic water use efficiency (WUEint; ratio Pn/gs) and instantaneous water use efficiency (WUEins; ratio Pn/E) were calculated with Pn, g_s_ and E according to Rahman et al. [33].

After exposure to NaCl and melatonin treatment for 15 days, leaves (0.3 g fresh weight) were quickly washed with ddH_2_O water and chlorophyll was extracted and measured according to the methods described by Liu et al. (2020). Briefly, leaves (0.3 g) were placed in a test tube with 5 mL 80% (v/v) formaldehyde and dimethyl sulfoxide and then bathed at 65 °C for 4 h. After cooling and filtration through filter paper, the filtrate was topped up to 5 mL with 80% acetone. The chlorophyll content was calculated by measuring the absorbance at 663 and 645 nm using a UV spectrophotometer (UV756, Shanghai Youke Co., Ltd.) (Liu et al. 2020).

### Measurements of H_2_O_2,_ O_2_^•–^, and malondialdehyde

The content of H_2_O_2_ and O_2_^•–^ in detached leaves was determined using a UV spectrophotometer (UV756, Shanghai Youke Co., Ltd.). H_2_O_2_ and titanium sulfate (or titanium chloride) form a yellow precipitate of peroxide titanium complex, which can be dissolved by H_2_SO_4_ (2 M) and determined by colorimetry at 415 nm. The O_2_^•–^ concentration was determined in plants by O_2_^•–^ hydroxylamine oxidation [14]. According to the above principle, the standard curve is generated with a standard H_2_O_2_ and O_2_^•–^ concentration gradient solution, and the regression curve between the H_2_O_2_ and O_2_^•–^ concentration and absorption value is obtained. The content of malondialdehyde (MDA) was measured according to Draper and Hadley [6] using the thiobarbituric acid method. Then, the same mass of plant tissue was ground with acetone and centrifuged at 1000 *g* and room temperature for 15 min to obtain the supernatant. The content of H_2_O_2_ and O_2_^•–^ under different melatonin treatments was observed directly in leaves by DAB and NBT staining under salt stress.

### Antioxidant activity assays

The activites of superoxide dismutase (SOD), catalase (CAT), peroxydase (POD), and ascorbate peroxidase (APX) were determined in the detached leaves of plants subjected to NaCl and melatonin treatments according to Li et al. [25].

### Transcriptome analysis

High-quality RNA was extracted from the samples and validated for transcriptome analysis. The quality of RNA extracted from the samples was analyzed by agarose gel electrophoresis and then verified (the RNA concentration, RIN value, 28S/18S and fragment size) using an Agilent 2100 Bio-analyzer (Agilent Technologies, Santa Clara, CA, United States). Then RNA purity (OD260/280) was detected using a ThermoFlyer NanoDrop UV spectrophotometer. The mRNA was isolated by Oligo-dT beads (Qiagen, Germany), and then randomly broken by adding the fragmentation buffer. Using random hexamers, first-strand cDNA was synthesized and then the second-strand cDNA using DNA polymerase I. The cDNA fragments were purified and enriched by PCR to construct the final RNA-Seq libraries. After the insert size of the libraries was checked and the concentrations quantified, the Illumina HiSeq platform was used for sequencing.

Raw RNA-Seq reads were filtered to remove adaptor sequences, repetitive sequences and low-quality reads (Quality value Phred ≤25 bases, accounting for more than 40% of total reads) and obtain clean, effective and high-quality sequences (clean reads). Clean reads were spliced with Trinity, and the spliced transcript sequences were used as reference sequences. Then the clean reads were mapped to the *L. bicolor* genome sequence using Hisat2 software for similarity analysis and then the genes or transcripts can be annotated and quantified. The *p*-value obtained from the original hypothesis test was corrected. Transcripts with *P*. adj < 0.05 and absolute log_2_foldchange value > 1 were considered to be significantly differentially expressed transcripts.

### qRT-PCR analysis

The nucleotide sequences of *L. bicolor* genes were obtained from the RNA sequences [54]. Primers were designed using Beacon Designer (Premier Biosoft, Palo Alto, California, USA) (Supplementary Table 1) and used to clone the conserved region sequences (about 800 bp) of genes that have been verified. Real-time PCR was performed using AceQ Universal SYBR Green qPCR Master Mix (Vazyme Biotech, Nanjing, China) and a real-time quantitative PCR instrument (Bio-Rad Laboratories, Hercules, California, USA). The relative expression of each gene was calculated using the 2^–△△Ct^ method [5, 14]. The housekeeping gene *LbTUBULIN* was used as an internal reference.

### Statistical analysis

The statistical analysis was performed using the SPSS software package (version 19.0; IBM, Armonk, New York, USA). The statistical significance was determined using an analysis of variance (ANOVA), and significant differences (*P* < 0.05) between the values were determined using Duncan’s multiple range test.

### Availability of data and materials

The data sets supporting the results of this article are included within the article and its additional files. The RNA-Seq raw reads produced in this study have been deposited in the National Center for Biotechnology Information (NCBI) Sequence Read Archive (SRA) under BioProject accession PRJNA758442, which includes 12 SRAs (SRR15682774, SRR15682775, SRR15682776, SRR15682777, SRR15682778, SRR15682779, SRR15682780, SRR15682781, SRR15682782, SRR15682783, SRR15682784, SRR15682785).

## Supplementary Information

**Additional file 1: Table S1.** The primers for genes used in real-time qPCR analysis.

**Additional file 2: Fig. S1.** The GO enriched functions of upregulated genes under melatonin+ 300 mM NaCl treatment compared to these under 300 mM NaCl treatment.

## Data Availability

Not applicable.

## Abbreviations

APX: ascorbate peroxidase
CAT: catalase
Ci: intercellular carbon dioxide concentration
g_s_: stomatal conductance
H_2_O_2_: hydrogen peroxide
*MAPK*: mitogen-activated protein kinase
MDA: malondialdehyde
O_2_^•–^: superoxide anion
Pn: net photosynthetic rate
POD: peroxidase
ROS: active oxygen species
SOD: superoxide dismutase
Tr: transpiration rate
