# Supplementary material for: Melatonin increases growth and salt tolerance of Limonium bicolor by improving photosynthetic and antioxidant capacity
Source: BMC Plant Biol. 2022 Jan 4;22:16. doi: 10.1186/s12870-021-03402-x (PMC8725383; doi:10.1186/s12870-021-03402-x)
Supplement: Supplementary file 1 — Additional file 1: Table S1. The primers for genes used in real-time qPCR analysis. [file 12870_2021_3402_MOESM1_ESM.docx]

**Supplementary Table 1.** The primers for genes used in real-time qPCR analysis.

| **Gene ID** | **F1 (5'**-**3')** | **F2 (5'**-**3')** |
| --- | --- | --- |
| *LbTUBULIN* | GGTTGAGTGAGCAGTTCAC | GATAACCAGCCACACCTTAGC |
| *LbMAPK* | CCTCCTATCATGCCAATC | CCTGCTCATTAGTCTCTG |
| *Lb0G37981* | AGTAGGATTAGGGCTAAAC | GTGCTGTGAAGAGAAATC |
| *Lb1G08035* | CTATCTTCAGCCTATATGC | GGTGTCCAGAGTAAGTAA |
| *Lb0G36726* | TGCCATTATTCCTACTTCC | CGCCATTGTATAACCATTC |
| *Lb4G25364* | GGCGTGGTTATTGGCAAGA | GCGTAGCAGGAGCAACTT |
| *Lb2G10556* | CTGTCGCCACTATTACTG | GTTGACGGAATACTGAAGA |
| *Lb3G20002* | CCTCTTCTTCTTCCTCAG | TTCCTCCACTCATCATTG |
| *Lb0G37481* | ATTGTTGGTCCTGTTCTCA | CGATGCTGCTGTCTATCT |
| *Lb1G07260* | AAGGCAACAGAAGAAGATG | GATGACGGAATCAGAAGAC |
| *Lb4G25364* | GGCGTGGTTATTGGCAAGA | GCGTAGCAGGAGCAACTT |
| *Lb0G37372* | GACCGAAGATTGGCACTC | GACATCGTAGGCACATTGG |
| *Lb1G04420* | CTTCCATCTCGTGTTATCTC | GAATCCATCACAATGTAGCA |
| *Lb0G37377* | TGATTGGTCTTCGTTCCT | AGCATCCGTCTCATAGTAAT |
| *Lb4G22624* | ATGTATCCACCGTTGTTG | CCTCTCCATCTCAGTCTT |
| *Lb1G00304* | TCTTCCAGTGCTAGTGTA | ATGTAATACCGTCCAGTTC |
