# Supplementary figures and images for: Melatonin increases growth and salt tolerance of Limonium bicolor by improving photosynthetic and antioxidant capacity
Source: BMC Plant Biol. 2022 Jan 4;22:16. doi: 10.1186/s12870-021-03402-x (PMC8725383; doi:10.1186/s12870-021-03402-x)

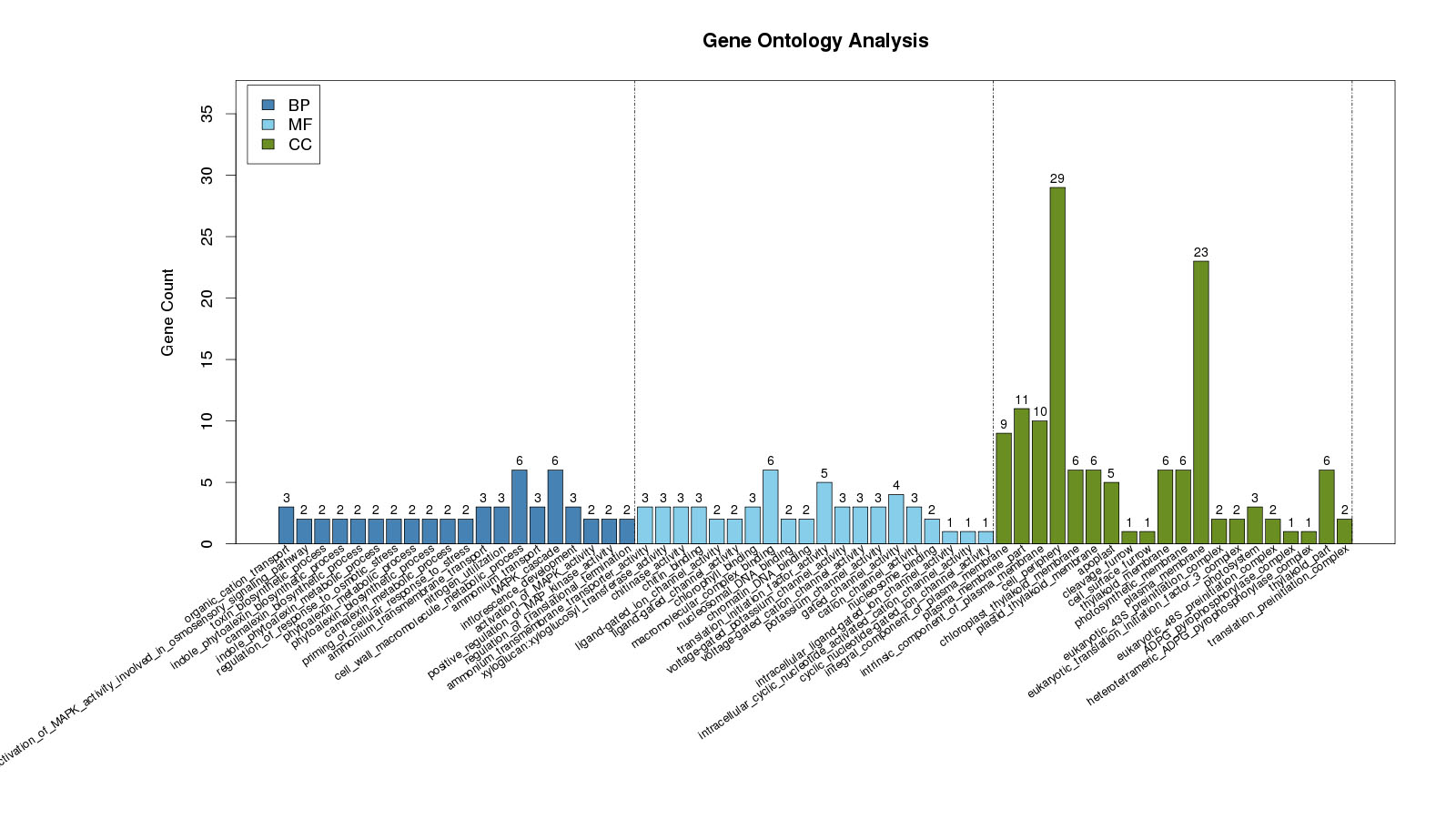

Supplement: Supplementary file 2 — Additional file 2: Fig. S1. The GO enriched functions of upregulated genes under melatonin+ 300 mM NaCl treatment compared to these under 300 mM NaCl treatment. [file 12870_2021_3402_MOESM2_ESM.jpg]
